# Mapping the state-of-the-art of the barriers for personalized preventive approaches worldwide: A scoping review of reviews

**DOI:** 10.1371/journal.pone.0335444

**Published:** 2025-10-24

**Authors:** Nicolò Scarsi, Abdelrahman Taha, Sara Farina, Tommaso Osti, Luigi Russo, Alessandra Maio, Roberta Pastorino, Stefania Boccia

**Affiliations:** 1 Department of Life Science and Public Health, Section of Hygiene, Università Cattolica del Sacro Cuore, Rome, Italy; 2 Department of Medicine and Surgery, University of Perugia, Perugia, Italy; 3 Department of Woman and Child Health and Public Health, Fondazione Policlinico Universitario A. Gemelli IRCCS, Rome, Italy; The First Hospital of Jilin University, CHINA

## Abstract

**Introduction:**

The growing prevalence of chronic diseases globally raised the public health need to improve the effectiveness of preventive medicine through the integration of big data, biological biomarkers and omics technologies. The implementation of personalized preventive approaches in real-world settings is constrained by several barriers. This scoping review aimed to map the barriers hindering the adoption of personalized preventive approaches for chronic diseases in health systems.

**Materials and Methods:**

PubMed, Web of Science, Scopus and gray literature sources were consulted from 2017 to 2024, to collect reviews on personalized preventive approaches implementation’s barriers. Additionally, we conducted a thematic analysis in order to categorize the identified barriers. The review followed Arksey-O’Malley guidelines and PRISMA-ScR checklist,

**Results:**

283 barriers were extracted from 37 reviews, and categorized into six main domains, namely “Research” (30 reviews), “Organizational Aspects” (27 reviews), “Healthcare Professionals” (28 reviews), “Ethical, Legal, and Social Issues” (29 reviews), “Public” (24 reviews), and “Financial concerns” (23 reviews). The research domain was characterized by lack of generalizability, clinical efficacy and cost effectiveness evidence. Organizational barriers included operational inefficiencies and unclear implementation frameworks. Healthcare professionals struggle with insufficient personalized prevention literacy. Ethical concerns, data privacy issues, and health inequities represent a burden to deal with for actual translation into practice, as well as the general mistrust of individuals and inadequate financial mechanisms,

**Conclusions:**

Our findings showed how several factors threaten the progress of many innovations in the field of personalized prevention across different populations and chronic diseases, highlighting the need for further efforts in personalized preventive approaches implementation.

## Introduction

Over the past two decades, significant advancements in life sciences and digital technologies have transformed our ability to develop tailored preventive interventions based on individual and environmental characteristics [1]. These advancements have been driven by the exponential decline in the costs of genome sequencing, fostering increased competition among sequencing companies, continuous innovation in genomics, and improvements in computational tools [2]. Together, these developments have accelerated the progress toward personalized prevention (PP) approaches, enabling more precise and effective strategies to maintain health and prevent disease [3]. As such approaches lie in the assumption to prevent and care for the right population and patients at the right time, the actual implementation of effective PP interventions in real world scenarios would in principle drive to lower healthcare related costs associated with the growing burden of chronic disease globally, which is expected to reach $47 trillion by 2030 [4]. Personalized preventive approaches leverage the use of biomarkers, including omics-based ones, to enable targeted preventive interventions across different stages of disease management, by predicting individual disease risk before onset (primary prevention), allowing early detection (secondary prevention), and estimating the likelihood of complications, recurrence or drug adverse events (tertiary prevention) [3]. To ensure effective and efficient implementation of clinically useful PP approaches, however, we should consider end users perspectives, (e.g., citizens’ and patients’ health literacy), healthcare workforce knowledge and awareness (e.g., physician education), as well as organizational, financial, legal, and ethical factors [5]. Understanding the barriers that impede the implementation of PP approaches is crucial for developing effective healthcare strategies to overcome them and to facilitate the successful translation of evidence into clinical practice [3,6]. Considering this, we conducted a scoping review of reviews to map the barriers that hinder the implementation of personalized preventive approaches within health systems using a global perspective.

## Materials and methods

The scoping review followed the 5-stage methodological framework described by Arksey and O’Malley [7]. The protocol has been uploaded to the Open Science Framework for public consultation, with registration DOI: https://doi.org/10.17605/OSF.IO/DBPHR.

### Search strategy and definitions

Using the launch of the International Consortium for Personalized Medicine in November 2016 as a key milestone, our search spanned from January 2017 to December 2024 to identify relevant reviews. We conducted searches across multiple databases including PubMed, Scopus, Web of Science, Google Scholar, and gray literature sources such as established networks in personalized medicine and genomics. Our search strategy used the following keywords: “personalized prevention”, “approach”, and “omics” (S1 File). For this search terms, we based on the consortium of the project “a PeRsonalised Prevention roadmap for the future HEalThcare” (PROPHET), which defined a *personalized preventive approach* as “an action, or a set of actions, in which the information provided by genetic and/or other omic biomarkers testing, combined with demographic, environmental and behavioral characteristics, socio-financial and cultural context of individuals, guides the decision- making process regarding one or more interventions aimed at preventing the onset, progression and recurrence of diseases” [3,6]. In this context, *omics technologies* are high-throughput analytical platforms that enable the comprehensive measurement and characterization of large sets of biological molecules, including genes, transcripts, proteins, metabolites, epigenetic modifications and microbiota composition. They represent a fundamental component of personalized prevention, as they support individual risk stratification by integrating genetic, molecular and environmental data, thereby facilitating tailored preventive strategies [8]. Additionally, we defined a “barrier” as any limitation, or obstacle to the implementation of personalized medicine approaches within health systems, encompassing laboratory and clinical research, health professionals’ and citizens’ knowledge, ethical, legal and social issues, and operational aspects.

### Eligibility criteria and assessment

To be included, reviews should meet the following criteria: any systematic, scoping, narrative review published in English language and primarily focused on barriers to the implementation of personalized preventive approaches. We excluded preprints, study protocols, primary studies, and literature reviews that did not primarily address barriers in personalized prevention. Eligible articles were uploaded in the Rayyan software [9], followed by a two-phase assessment to determine eligibility: titles and abstracts’ screening, and full-text review. We piloted the screening process and data extraction, conducting them in a double-blind fashion by three researchers (NS, AT, SF) to ensure consistency and resolve any potential disagreements.

### Data extraction

From each eligible document, we extracted information including first author, year of publication, country where the study was conducted, and any barrier identified in the included reviews. Furthermore, for each identified barrier, we extracted the health condition or disease and the omics- based technology used.

### Data synthesis

To categorize the extracted barriers, we conducted a qualitative content analysis of the textual transcriptions using thematic analysis, which we implemented through three phases. In the first phase, we read the textual transcriptions or synthetic descriptions of the barriers to establish preliminary impressions among the team. In the second phase, we developed a coding framework, defining and naming different themes and subthemes. Finally, in the third phase, team members discussed the findings and reached a consensus on the final thematic structure, categorizing the identified barriers into major barrier domains. We computed descriptive statistics for each domain and subdomains and presented the results narratively, delineating them in tables.

### Reporting

The review followed the Preferred Reporting Items for Systematic reviews and Meta-Analyses extension for Scoping Reviews (PRISMA-ScR) Checklist (S2 File) [10].

## Results

**Fig 1:**
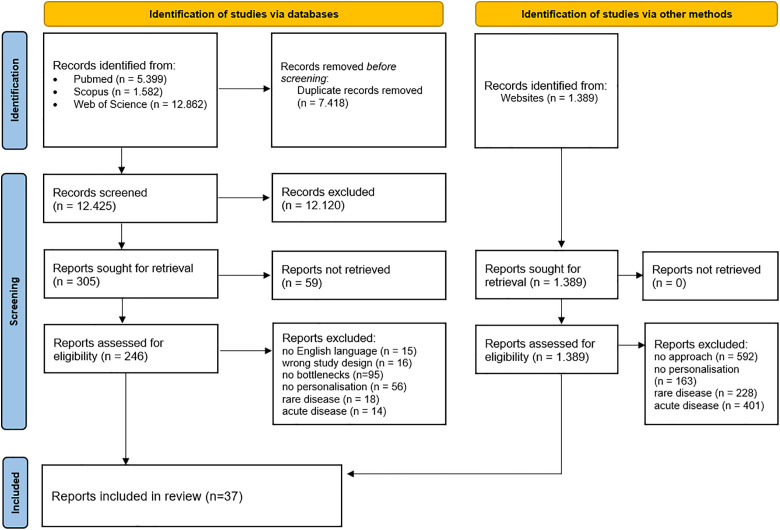
PRISMA flow chart for study selection.

Of the 12,425 unique records identified through database searching, we assessed 246 articles for full-text eligibility and included 37 reviews (Fig 1). Among these, 16 studies (43%) addressed barriers regarding chronic diseases in general, while the remaining 21 focused on specific disease-related barriers. Specifically, among these, 9 (24%) were on cancer (e.g., breast, colorectal, hepatocellular cancers), 5 (14%) on cardiovascular diseases (e.g., myocardial infarction), 4 (11%) on metabolic disorders (e.g., obesity, diabetes), and 3 (8%) on neurological and psychiatric disorders (e.g., Alzheimer). Concerning the omics technology, 28 reviews (76%) were focused on genetic or genomic testing, 4 (11%) on multi-omics, 3 (8%) on pharmacogenomics, and only 2 (5%) were on metabolomics.

### Thematic analysis

From the 37 reviews we extracted overall 283 barriers (S1 Table), summarized into six major domains from the thematic analysis: “*Research”, “Organizational aspects”, “Healthcare professionals”, “Ethical, Legal, and Social Issues (ELSI)”, “Public”, and “Financial concerns”*. The definition given for each domain is reported below:

- *Research* includes factors that impede scientific advancement and robust evidence generation.
- O*rganizational aspects* focus on challenges in the integration of personalized approaches into healthcare and the practical translation of personalized prevention in real-world context.
- The *Healthcare Professionals* domain covers issues like limited training in the field, and workload concerns.
- The *Ethical, Legal, Social Issues (ELSI)* domain encompasses ethical and legal concerns, including privacy, consent, and equity.
- *Public* addresses barriers regarding citizens and patients, including limited health literacy and skepticism toward personalized prevention.
- Lastly, the *Financial concerns* domain includes economic hurdles, such as high costs and funding limitations for the implementation of personalized prevention approaches.

The description of the barriers retrieved in the scoping review according to domains and subdomains is provided in the following paragraphs, along with details of the disease and the main omics technology included.

### Research

Thirty reviews (81%) identified research as a barrier for implementation of personalized prevention [11–40]**.** In this specific domain, we identified 5 different subdomains, namely lack of evidence across different ethnicities, lack of clinical efficacy evidence, lack of clinical validity evidence, lack of cost-effectiveness evidence, and lack of analytical validity evidence (Table 1). The most prevalent barrier was the generalizability and applicability of findings across different ethnicities, found in 18 reviews (60%) [11–28]. Of these, 3 reviews focused on cancer (17%), 3 on cardiovascular diseases (17%), 2 on neurological and psychiatric disorders (11%), 2 on metabolic disorders (11%), and 8 on chronic diseases in general (44%). Additionally, 72% of the reviews addressing this barrier referred to genetic/genomic testing, and among these two focused on the transferability of polygenic risk score (PRS) to different ethnic groups, due to the underrepresentation in validation studies and the potential variability of PRS performance across different populations.

**Table 1:** Characteristics of 30 reviews on research barriers according to subdomain, disease type, and omics technology.

Subdomains	N. of reviews (%)*	Disease (%)	Omics (%)	Reference
***Lack of evidence across different ethnicities***	18 (60%)	Cancer 3 (17%) Cardiovascular diseases 3 (17%) Neurological/psychiatric disorders 2 (11%) Metabolic disorders 2 (11%) Chronic diseases 8 (44%)	Genetic/genomic testing 13 (72%) Pharmacogenomics 2 (11%) Multi-omics 2 (11%) Metabolomics 1 (6%)	11-28
***Lack of clinical efficacy evidence***	16 (53%)	Cancer 5 (31%) Cardiovascular diseases 2 (13%) Neurological/psychiatric disorders 2 (13%) Metabolic disorders 1 (6%) Chronic diseases 6 (37%)	Genetic/genomic testing 13 (81%) Pharmacogenomics 2 (13%) Multi-omics 1 (6%)	14-21;31-38
***Lack of clinical validity evidence***	15 (50%)	Cancer 5 (33%) Metabolic disorders 2 (13%) Cardiovascular diseases 1 (7%) Chronic diseases 7 (47%)	Genetic/genomic testing 11 (73%) Metabolomics 2 (13%) Multi-omics 2 (13%)	15-20;24-32
***Lack of cost effectiveness evidence***	8 (27%)	Cancer 3 (37%) Cardiovascular diseases 2 (25%) Chronic diseases 3 (37%)	Genetic/genomic testing 5 (63%) Pharmacogenomics 2 (25%) Multi-omics 1 (12%)	19-22;37-40
***Lack of analytical validity evidence***	5 (17%)	Cancer 2 (40%) Cardiovascular diseases 1 (20%) Metabolic disorders 1 (20%) Chronic diseases 1 (20%)	Genetic/genomic testing 3 (60%) Multi-omics 2 (40%)	18-20;23;40

*The percentages of reviews for each subdomain are calculated based on a total of 30 reviews that include barriers related to research. The total does not add up to 100% because a single review may address more than one subdomain, and each subdomain may be cited by more than one review.

Consistently, 16 reviews (53%) reported the lack of clinical efficacy evidence as a barrier in personalized prevention, along several different disease types (Table 1) [14–21,29–36]. Concerning the omics technology, 13 reviews (81%) concentrated on genetic/genomic testing, while 2 reviews (13%) focused on pharmacogenomics and one (6%) included a multi-omics approach. Recurring barriers included issues in designing robust, large scale randomized clinical trials in supporting clinical efficacy of the preventive intervention following a genetic test. For instance, one study noted limited clinical efficacy data supporting PRS for breast cancer prevention, while another highlighted the insufficient evidence of clinical efficacy for apolipoprotein E (ApoE) genotyping in assessing the risk of late onset Alzheimer disease.

Fifteen reviews (46%) reported barriers related to the lack of clinical validity evidence of omics technologies in cancer (33%), metabolic disorders (13%), cardiovascular diseases (7%), and chronic diseases in general (47%) [15–20,24–30,37,38]. Among these, the majority (73%) were on genetic/genomic testing. The major drawbacks reported concerned the limited evidence of robust and validated biomarkers, diagnostic tools, and genetic variants.

Eight reviews (27%) highlighted the lack of cost effectiveness analyses across cancer (37%), cardiovascular diseases (25%) and chronic diseases in general (37%), applied in the field of genetic/genomic (63%), pharmacogenomic (25%) and multi-omics (12%) testing [19–22,35,36,39,40].

Five reviews (17%) discussed the lack of analytical validity evidence in personalized prevention, highlighting the challenges in identifying DNA alterations and the lack of evidence to develop analytical infrastructures, able to handle multi-omics data in the treatment of various chronic diseases [18–20,23,40].

### Organizational aspects

A total of 27 reviews (73%) mentioned barriers related to the organizational aspects of personalized preventive approaches [11,12,14,16–21,24–29,37,38,31–33,35,36,39–43]**.** Three subdomains were identified, namely operations and logistics, lack of guidelines/standards for omics tests implementation, and policy makers poor knowledge (Table 2).

**Table 2:** Characteristics of 27 reviews on organizational barriers according to subdomain, disease type, and omics technology.

Subdomains	N. of reviews (%)*	Disease (%)	Omics (%)	Reference
***Operations and logistics***	25 (93%)	Cancer 5 (20%) Cardiovascular diseases 4 (16%) Neurological/psychiatric disorders 2 (8%) Metabolic disorders 2 (8%) Chronic diseases 12 (48%)	Genetic/genomic testing 18 (72%) Pharmacogenomics 3 (12%) Metabolomics 2 (8%) Multi-omics 2 (8%)	11,12,14,16-21,24-31,34,37-43
***Lack of guidelines/standards for omics tests implementation***	11 (41%)	Cancer 4 (36%) Cardiovascular diseases 1 (9%) Neurological/psychiatric disorders 1 (9%) Chronic diseases 5 (46%)	Genetic/genomic testing 9 (82%) Metabolomics 1 (9%) Multi-omics 1 (9%)	17-20,24,29, 31,33,35,37,40
***Policy makers poor knowledge***	3 (11%)	Cancer 1 (33%) Cardiovascular diseases 1 (33%) Chronic diseases 1 (33%)	Genetic/genomic testing 3 (100%)	35,37,40

*The percentages of reviews for each subdomain are calculated based on a total of 27 reviews that include organizational barriers. The total does not add up to 100% because a single review may address more than one subdomain, and each subdomain may be cited by more than one review.

Twenty-five reviews (93%) acknowledged the presence of operations and logistics constraints, mostly focusing on cancer (20%), cardiovascular diseases (16%), neurological and psychiatric disorders (8%), and metabolic disorders (8%), while 12 were on chronic diseases in general (48%) [11,12,14,16–21,24–29,37,38,32,35,43]. Most of the reviews in this subdomain (72%) concerned genetic/genomic testing, recurrently highlighting issues of data management, interoperability, and integration, especially with data from other omics sources, including metabolomics and proteomics.

Eleven reviews (41%) discussed the lack of guidelines or standards for -omics tests implementation, of which 4 were on cancer (36%), one on cardiovascular diseases (9%), one on neurological and psychiatric disorders (9%) and 5 on chronic diseases in general (46%) [17–20,24,37,29,31,33,35,40]. In relation to omics technologies, these included genetic/genomic testing (82%), metabolomics (9%) and multi-omics approaches (9%). The main issues recognized were the absence of professional guidelines and legal standard of care, and the lack of regulatory aspects about sample collection, data storage, and data sharing.

Lastly, 3 reviews complained about the poor awareness among policymakers of the potential benefits and challenges of genomic medicine use for chronic diseases prevention, including cancer and cardiovascular diseases [33,35,40].

### Healthcare professionals

A total of 28 reviews (76%) mentioned barriers linked to the healthcare professionals [11,16–22,24–28,29–36,38–41,43–45], where we identified three subdomains, namely poor knowledge from healthcare professionals, absence of specialized healthcare professionals, and limited acceptance or belief from healthcare professionals (Table 3). The most prevalent barrier pertained to the poor knowledge from healthcare professionals, reported in 27 reviews (96%). Of these, 8 (30%) were on cancer, 4 (15%) on cardiovascular diseases, 3 (11%) on metabolic disorders, 2 (7%) on neurological and psychiatric disorders, and 10 (37%) on chronic diseases in general. Most reviews were about genetic/genomic testing (82%) and discussed the lack of awareness and education among doctors and other healthcare professionals, that often results in misunderstandings of genomic findings and inadequate translation of discoveries into recommendations for patients.

**Table 3:** Characteristics of 28 reviews on healthcare professionals’ barriers according to subdomain, disease type, and omics technology.

Subdomains	N. of reviews (%)*	Disease (%)	Omics (%)	Reference
***Poor knowledge of healthcare professionals***	27 (96%)	Cancer 8 (30%) Cardiovascular diseases 4 (15%) Metabolic disorders 3 (11%) Neurological/psychiatric disorders 2 (7%) Chronic diseases 10 (37%)	Genetic/genomic testing 22 (82%) Pharmacogenomics 2 (7%) Multi-omics 2 (7%) Metabolomics 1 (4%)	11,16-22,24-28, 30-41,43.45
***Absence of specialized healthcare professionals***	7 (25%)	Cardiovascular diseases 2 (29%) Cancer 1 (14%) Neurological/psychiatric disorders 1 (14%) Chronic disease 3 (43%)	Genetic/genomic testing 6 (86%) Multi-omics 1 (14%)	11,18-20,22,35,41
***Limited acceptance or belief from healthcare professionals***	6 (21%)	Cardiovascular diseases 2 (33%) Cancer 1 (17%) Neurological/psychiatric disorders 1 (17%) Chronic diseases 2 (33%)	Genetic/genomic testing 5 (83%) Pharmacogenomics 1 (17%)	18,20,21,27,33,43

*The percentages of reviews for each subdomain are calculated based on a total of 28 reviews that include healthcare professionals’ barriers related to research. The total does not add up to 100% because a single review may address more than one subdomain, and each subdomain may be cited by more than one review.

Seven reviews (25%) reported the absence of specialized healthcare professionals mostly in genetics and genomics, of which 2 (29%) were on cardiovascular diseases, one (14%) on cancer, one (14%) on neurological and psychiatric disorders and 3 on chronic diseases in general, pointing out the limited availability of genetic counseling services [11,18–20,22,33,41].

Lastly, 6 reviews (21%) mentioned the limited acceptance or belief from healthcare professionals, of which 2 were on cardiovascular diseases, one on cancer, one on neurological and psychiatric disorders, and 2 on chronic diseases in general [18,20,21,27,31,43]. About the omics technology, 5 (83%) reviews were on genetic/genomic testing while only one on pharmacogenomics. Among the factors analyzed, low attitudes of clinicians towards genomics were observed, including the limited awareness and acceptability of genetic testing, along with the perception that omics lies outside some providers’ areas of expertise.

### Ethical, legal and social issues

Twenty-nine reviews (78%) mentioned ELSI barriers related to the implementation of personalized preventive approaches [11–14,16–20,22,24,25,27–30,37,38,32,33,35,39–46]. Three subdomains were identified, namely data sharing and privacy issues, health provision inequalities/accessibility/social disparities, and inequalities between countries (Table 4). Major barriers concentrated on data sharing and privacy issues, reported in 23 (77%) reviews across several diseases (Table 4). Concerning the omics technology, 20 reviews (87%) were about genetic/genomic testing, while the remaining 3 were on metabolomics and multi-omics [14,16–20,24,25,27–30,37,38,32,33,35,39–43,45]. Overall, the issues described pertain to the regulatory aspects regarding different fields, including data availability and management across different sectors, and the integration of new technologies into clinical practice, arising concerns about the use by third parties.

**Table 4:** Characteristics of 29 reviews on ethical, legal and social issues (ELSI) barriers according to subdomain, disease type, and omics technology.

Subdomains	N. of reviews (%)*	Disease (%)	Omics (%)	Reference
***Data sharing and privacy issues***	23 (77%)	Cancer 7 (30%) Cardiovascular diseases 2 (9%) Neurological/psychiatric disorders 1 (4%) Metabolic disorders 1 (4%) Chronic diseases 12 (52%)	Genetic/genomic testing 20 (87%) Multi-omics 2 (9%) Metabolomics 1 (4%)	14,16-20,24,25, 27-32,34,35,37, 39-43,45
***Health provision inequalities, accessibility and social disparities***	18 (60%)	Cancer 7 (39%) Cardiovascular diseases 3 (17%) Neurological/psychiatric disorders 1 (5%) Chronic diseases 7 (39%)	Genetic/genomic testing 16 (88%) Pharmacogenomics 1 (6%) Multi-omics 1 (6%)	11-13,17-20, 22,27,31,32,35, 39-42,44,46
***Inequalities between countries***	3 (4%)	Cancer 1 (33%) Cardiovascular diseases 1 (33%) Neurological/psychiatric disorders 1 (33%)	Genetic genomic testing 3 (100%)	11,18,20

*The percentages of reviews for each subdomain are calculated based on a total of 29 reviews that include ELSI barriers. The total does not add up to 100% because a single review may address more than one subdomain, and each subdomain may be cited by more than one review.

Eighteen reviews (60%) addressed problems such as health provision inequalities, accessibility and social disparities, of which 7 were on cancer (39%), 3 on cardiovascular diseases (17%), one on neurological and psychiatric disorders, and 7 on chronic diseases in general [11–13,17–20,22,27,29,30,33,39–42,44,46]. Almost all reviews (88%) focused on genetics/genomics, except of one centered on pharmacogenomics and one on multi-omics, and addressed significant inequities concerns in the provision of care, and how the differential access and the benefits of precision medicine may exacerbate social disparities across different socio economics, racial, or ethnic groups. Lastly, 3 reviews focused on inequalities between low-, middle-, and high-income countries, in the scope of genetic/genomic testing for cancer, cardiovascular diseases and neurological and psychiatric disorders [11,18,20].

### Public

Twenty-four reviews (65%) mentioned individuals related barriers to the implementation of personalized preventive approaches [11,12,14,16–20,24–26,28,30,32,33,35,39–43,45–47]. Six subdomains were identified, namely discrimination, psychological impact of results, poor education, family, lack of trust and belief in personal benefit, willingness to research participation (Table 5). Most prevalent issues regarded the potential risk of discrimination upon genetic risk results, which has been reported in 18 reviews (75%) [11–14,18–20,24,30,32,33,35,39–42,45,47]. Of these, 5 were on cancer, 3 on cardiovascular diseases, 2 on neurological and psychiatric disorders, and 8 on chronic diseases in general. Most of the reviews (83%) concerned genetic/genomic testing, highlighting concerns potentially linked to cultural disrespect, insurance, work environment, religion, and other negative social effects, especially in vulnerable populations. One study specifically pointed out the ethical aspects surrounding the use of PRS, as a potential aggravating factor of discrimination.

**Table 5:** Characteristics of 24 reviews on public related barriers according to subdomain, disease type, and omics technology.

Subdomains	N. of reviews (%)*	Disease (%)	Omics (%)	Reference
***Discrimination***	18 (75%)	Cancer 5 (28%) Cardiovascular diseases 3 (17%) Neurological/psychiatric disorders 2 (11%) Chronic diseases 8 (44%)	Genetic/Genomic testing 15 (83%) Multi-omics 2 (11%) Pharmacogenomics 1 (6%)	11-14,15-20, 24,32,34,35,37, 39-43,45,47
***Psychological impact of results***	12 (50%)	Cancer 4 (33%) Neurological/psychiatric disorders 2 (17%) Cardiovascular diseases 1 (8%) Chronic diseases 5 (42%)	Genetic/Genomic testing 11(92%) Multi-omics 1 (8%)	11,14,17,18,24,34, 35,39,40,42,45,47
***Poor education***	11 (46%)	Cancer 3 (27%) Cardiovascular diseases 1 (9%) Metabolic disorders 1 (9%) Chronic disease 6 (55%)	Genetic genomic testing 8 (73%) Multi-omics 2 (18%) Metabolomics 1 (9%)	16,19,26,28,32, 37,39,42,43,45,47
***Family***	9 (37%)	Cancer 1 (11%) Cardiovascular diseases 1 (11%) Neurological/psychiatric disorders 1 (11%) Chronic diseases 6 (67%)	Genetic/Genomic testing 9 (100%)	11,24,25,34, 35,42,43,45,46
***Lack of trust and belief in personal benefit***	8 (33%)	Cardiovascular diseases 2 (25%) Neurological/psychiatric disorders 1 (12%) Chronic diseases 5 (63%)	Genetic/Genomic testing 5 (63%) Multi-omics 2 (25%) Pharmacogenomics 1 (12%)	11,12,18,19, 34,37,42,47
***Willingness to research*** ***participation***	7 (29%)	Cancer 1 (14%) Cardiovascular diseases 1 (14%) Chronic diseases 5 (72%)	Genetic/Genomic testing 6 (86%) Multi-omics 1 (14%)	18,20,28, 34,42,46,47

*The percentages of reviews for each subdomain are calculated based on a total of 24 reviews that include public related barriers. The total does not add up to 100% because a single review may address more than one subdomain, and each subdomain may be cited by more than one review.

Twelve reviews (50%) mentioned the potential negative psychological impact of results on patients, in the scope of cancer (33%), neurological and psychiatric disorders (17%), cardiovascular diseases (8%) and chronic diseases in general (42%) [11,14,17,18,24,32,33,39,40,42,45,47]. All except one focused on genetic/genomic testing, mostly discussing the negative impact of personal genetic risk communication on self-perception and psychological issues, including stress, anxiety and depression. For instance, one review mentioned the emotional burden associated with providing genetic risk information for a disease with no effective treatment, as the case of ApoE genotyping for Alzheimer disease.

Eleven reviews (46%) reported barriers to the poor literacy on omics among citizens or patients, with respect to cancer (27%), cardiovascular diseases (9%), metabolic disorders (9%) and chronic diseases in general (55%) [16,19,26,28,30,35,42,43,45,47]. The majority of them (73%) were on genetic/genomic testing and underlined the lack of understanding and awareness of genetic/genomic technologies implications. One study, instead, specifically focusing on issues in understanding the potential benefit of PRS applications in breast cancer and two studies on the lack of knowledge of familial hypercholesterolemia (FH), respectively.

Nine reviews (37%) highlighted family related issues, in the case of cancer (11%), cardiovascular diseases (11%), neurological and psychiatric disorders (11%), and chronic diseases in general (67%), with all of them focusing on genetic/genomic testing [11,24,25,32,33,42,43,45,46]. Testing may accompany potentially sensitive family issues, causing troubles linked to the communication of genetic risk information to family members, misunderstandings, and lack of family involvement in the decision-making process.

Eight reviews (33%) reported a lack of trust and belief in personal benefit across cardiovascular diseases, neurological and psychiatric disorders and chronic diseases in general [11,12,18,19,32,35,42,47]. Among these, 5 reviews were on genetic/genomic testing, 2 on multi-omics, and one on pharmacogenomics (Table 3). Most reviews of this subdomain mentioned the mistrust of citizens and patients in the utility and necessity of getting tested, due to their poor knowledge and literacy about the potential personal benefit of personalized interventions. Lastly, 7 reviews (29%) pointed out issues related to the willingness of research participation, of which one was on cancer (14%), one on cardiovascular diseases (14%) and 5 were on chronic diseases in general (72%) [18,19,28,32,42,46,47]. The almost totality (86%) were on genetic/genomic testing and mentioned the lack of sufficient patients’ awareness and engagement as a barrier in research conduction in the field of genomics.

### Financial concerns

A total of 23 reviews (62%) mentioned financial barriers related to the implementation of personalized preventive approaches, and three subdomains were identified, namely high technology costs (including insurance coverage and reimbursement mechanisms), lack of standards and guidelines related to reimbursement mechanisms, and lack of resources (Table 6) [11,14,16–20,22,24,26–29,37,38,31,33,35,39,41–43,46]. Most prevalent issues pertained to the high technology costs, including insurance coverage and reimbursement mechanisms, reported in 20 reviews (87%) across different diseases (Table 6) [11,14,16–20,22,24,26–28,37,29,32,35,39,42,43,46]. Concerning the omics technology, the vast majority were about genetic/genomic testing (85%). For instance, high cost of mass spectrometry and nuclear magnetic resonance spectroscopy have been reported in metabolomics approaches, as well as for Next Generation Sequencing, ApoE genotyping, and testing costs for common FH-associated pathogenic, which remain significant.

**Table 6:** Characteristics of 23 reviews on financial concerns according to subdomain, disease type, and omics technology.

Subdomains	N. of reviews (%)*	Disease (%)	Omics (%)	Reference
***High technologies costs, including insurance coverage and reimbursement mechanisms***	20 (87%)	Cancer 4 (20%) Cardiovascular diseases 3 (15%) Neurological/psychiatric disorders 2 (10%) Metabolic disorders 1 (5%) Chronic diseases 10 (50%)	Genetic/genomic testing 17 (85%) Metabolomics 2 (10%) Multi-omics 1 (5%)	11,14,16-20,22, 24,26-29,31,34, 37,39,42,43,46
***Lack of guidelines/standards related to reimbursement mechanisms***	10 (43%)	Cancer 2 (20%) Cardiovascular diseases 1 (10%) Metabolic diseases 1 (10%) Chronic diseases 6 (60%)	Genetic/genomic testing 8 (80%) Multi-omics 2 (20%)	18-20,24,28, 30,31,35,37,41
***Lack of resources***	3 (13%)	Cancer 2 (66%) Cardiovascular diseases 1 (33%)	Genetic/genomic testing 3 (100%)	18,20,31

*The percentages of reviews for each subdomain are calculated based on a total of 23 reviews that include financial barriers. The total does not add up to 100% because a single review may address more than one subdomain, and each subdomain may be cited by more than one review.

Ten reviews (43%) mentioned the lack of standards and guidelines related to reimbursement mechanisms, of which 2 were on cancer, one on cardiovascular diseases, one on metabolic diseases and 6 on chronic diseases in general, the majority (80%) focused on genetic/genomic testing [18–20,24,28,38,29,33,35,41]. The main issues were about health regulation and commercialization and the lack of reimbursement policies, affecting the availability, affordability and access of genomic testing. Lastly, only 3 reviews (13%) reported the lack of resources as a barrier, focusing on lack of decision support teams and economic resources for genetic/genomic testing for cancer and cardiovascular diseases [18,20,21].

## Discussion

This scoping review synthesizes findings from 37 studies, offering a comprehensive overview of the barriers to the implementation of personalized preventive approaches for chronic diseases across multiple domains: research, organizational aspects, healthcare professionals, ELSI issues, public and financial constraints. The search highlighted that half of the studies were published in the US, with significant contribution from countries worldwide (including UK, Netherlands, Portugal, Spain, Italy, Germany, Austria, Mexico, Canada, India, Japan), reflecting the growing interest of the global scientific community towards this field over the timeframe considered.

Research-related barriers remain among the most pressing issues, with 81% of studies highlighting a lack of robust evidence, that hamper PP approaches implementation. A major concern is the overrepresentation of populations of European ancestry in genomic studies, which limits the generalizability of findings to diverse populations. This imbalance exacerbates health inequities and hinders the global applicability of genomic tools, as demonstrated by the inconsistent performance of PRS across different ethnic groups [48,49]. Nonetheless, significant progress has been made in recent years. Validation studies focusing on underrepresented ethnic groups have assessed the applicability of PRS, while advancements in multi-ancestry data analysis and models addressing ancestral-differential effects have markedly improved the accuracy of genomic predictions across diverse populations, helping to address the historical bias toward European ancestry in genomic research [49].

Additionally, there is a lack of clinical efficacy evidence to support the widespread implementation of personalized preventive approaches. Given the complexity of these interventions, exploring alternative methodologies and study designs, such as simulation models, may potentially provide valuable insights, especially as traditional RCTs with long-term follow-up can be logistically challenging and resource intensive [50–52].

Furthermore, organizational barriers and ELSI aspects were particularly prominent, identified in 78% of the included reviews. Issues related to data management, integration, and sharing emerged as significant obstacles. These challenges hinder collaboration among stakeholders, including clinicians, researchers, policymakers, and healthcare providers, as well as across countries and institutions. An essential effort involves regulating data sharing practices that are both sustainable and aligned with privacy protection standards. This includes not only health data, but also non-health data collected from personal devices and private companies [53,54]. Promising initiatives, such as the European Health Data Space, aim to address these challenges by creating a secure platform for international health data exchange within the EU. Such efforts facilitate the use of real-world data to generate evidence that supports personalized medicine and improves healthcare outcomes. ELSI-related barriers also highlight the risk of inequality and discrimination due to unequal access to personalized prevention, consistent with the financial constraints identified in the review. In universal healthcare systems, limited resources and the inability to provide advanced personalized services to all patients can lead to long waiting lists and uneven access, especially in rural areas [55]. In insurance-based systems, disparities are often driven by insurance coverage, with patients holding private insurance having better access to precision medicine [56]. In both systems, the lack of uniform coverage for genetic tests may also drive the use of direct-to-consumer tests, which are often unvalidated and can cause unnecessary anxiety or false reassurance [57]. Therefore, it is crucial to regulate reimbursement mechanisms and uniform insurance coverage to ensure equitable access to these services and minimize disparities in healthcare delivery.

In addition to the barriers already outlined, it is crucial for healthcare professionals to enhance their curricula regarding the use of omics technologies. Such improvements are essential to foster greater trust and acceptance of personalized prevention strategies while minimizing the potential for misinterpretation of findings.

Patients and citizens must be placed at the center of genomic initiatives. Achieving this requires us to eliminate the risk of discrimination based on genetic risk results, address the psychological and emotional stressors that individuals face, enhance genomic literacy, and actively engage with their families, relatives, and probands. However, findings from a recent review by Kreeftenberg LL et al. reveal that many initiatives aimed at involving patients and the public in personalized prevention through genomic information often demonstrate lower levels of engagement [58].

The majority of the identified and discussed barriers are related to the implementation of personalized prevention for oncology. This is likely due to the greater interest of the scientific community in this field and the higher availability of studies and data [59]. Exceptions include barriers associated with healthcare professionals, such as limited acceptance or belief and absence of specialized professionals, as well as public-related barriers, including lack of trust and belief in personal benefit and willingness to research participation, which show a higher prevalence in the context of neurodegenerative and psychiatric diseases. This may be due to the unique challenges posed by these conditions, including the complexity of genomic findings, stigmatization, and the psychological burden associated with predictive information [60].

However, it is important to highlight that most of the barriers identified in the reviews are related to the general challenges of implementing personalized prevention for chronic diseases. This highlights that many of the barriers are comparable and transferable across different diseases

Genomics emerged as the technology most frequently associated with barriers across all domains and subdomains, compared to other less-studied omics fields. This does not necessarily mean that genomics faces more challenges in integrating personalized prevention approaches. Instead, it likely reflects its wider implementation, resulting in a larger volume of data and examples to analyze. Moreover, the focus on genomics over the past two decades has produced a greater body of scientific literature compared to other omics disciplines. While our review did not specifically emphasize this, many barriers, such as those related to limited clinical efficacy evidence, insufficient knowledge among healthcare professionals, regulatory gaps, and high costs, are common across all omics fields, not just genomics. Overall, our thematic analysis revealed several cross-cutting subthemes that transcend individual domains and shape the broader landscape of barriers to personalized prevention. For example, the lack of robust evidence, whether related to clinical validity, cost-effectiveness, or generalizability, emerged not only as a research barrier but also influenced professional confidence, public trust, and policy decisions. Gaps in data governance and interoperability were found at the intersection of organizational and ELSI barriers, with direct implications for equity, cross-border collaboration, and sustainability. Financial constraints interacted with these issues, limiting the generation of high-quality evidence, the training of specialized professionals, and the equitable provision of services.

What is already known is that these interconnected barriers must be addressed simultaneously, as progress in one area, such as evidence generation, depends on advances in others, such as data infrastructure, workforce capacity, and equitable access. What remains to be understood is how to operationalize this alignment across diverse health systems, socioeconomic contexts, and cultural settings, and how to integrate multi-omics data in ways that reduce rather than widen existing inequalities.

By synthesizing the tabular findings into these interconnected themes, our review offers a structured map of the current state of barriers, consistent with the aim expressed in the title, and identifies priority areas where research and policy interventions can most effectively accelerate the implementation of personalized prevention.

Findings of our study are comparable with key issues identified and discussed during three Country Exchange Visits (CEVs) in the scope of Beyond 1 Million Genomes (B1MG) project, and synthesized by Lopes et al. [61]. These are: a) Patient and citizens trust and engagement; b) Infrastructure for implementation of genomics in healthcare practice; c) Ethical and legal frameworks; d) Synergies among healthcare, research and industry; e) Training of healthcare professionals. Overall, these claimed factors must be addressed to build efficient and sustainable genomic medicine strategies worldwide. Taken together, all the barriers identified in this scoping review can contribute to inform policy makers in ensuring a safe translational process of genomics in the future healthcare [61].

Our scoping review has some limitations that should be recognized. Firstly, as a scoping review of reviews, its objective is to map the barriers that hinder the proper implementation of personalized preventive approaches in health systems, without assessing the quality of included studies or evaluating the effect size of the retrieved barriers. Consequently, we cannot provide recommendations for healthcare professionals or policymakers, as this research was not aimed at gathering facilitators nor propose solutions to overcome the barriers identified. Secondly, the review may not be exhaustive of all known barriers to the implementation of personalized prevention, across several diseases, approaches, and countries. Lastly, the eligibility criteria constrained the search to records published in English between 2017 and 2024, potentially missing relevant analysis in the field.

In conclusion, the implementation of personalized preventive approaches faces numerous barriers, ranging from scientific and technological challenges to organizational, social and financial constraints. Addressing these obstacles requires coordinated efforts across multiple levels. It is essential to foster inclusive research frameworks, strengthen professional education, and develop policies that promote equity and accessibility, to ensure that all populations can benefit from these innovations, reducing health disparities and enhancing the effectiveness of prevention strategies globally.

By overcoming these challenges, healthcare systems can unlock the full potential of personalized prevention, enabling more tailored and impactful interventions that improve outcomes for diverse patient populations.

## Supporting information

S1 File Search strategies for scientific databases and grey literature. (DOCX)

S2 File Preferred Reporting Items for Systematic reviews and Meta-Analyses extension for Scoping Reviews (PRISMA-ScR) Checklist. (DOCX)

S1 Table Detailed overview of identified barriers. (XLSX)
