# Supplementary material for: Mapping the state-of-the-art of the barriers for personalized preventive approaches worldwide: A scoping review of reviews
Source: PLoS One. 2025 Oct 24;20(10):e0335444. doi: 10.1371/journal.pone.0335444 (PMC12551886; doi:10.1371/journal.pone.0335444)
Supplement: S1 File — (DOCX) [file pone.0335444.s001.docx]

**S1 File. Search strategies for scientific databases and grey literature.**

**Search strategy for PubMed:**

(("personal prevention"[All Fields] OR "individual prevention"[All Fields] OR (("predict"[All Fields] OR "predictabilities"[All Fields] OR "predictability"[All Fields] OR "predictable"[All Fields] OR "predictably"[All Fields] OR "predicted"[All Fields] OR "predicting"[All Fields] OR "prediction"[All Fields] OR "predictions"[All Fields] OR "predictive"[All Fields] OR "predictively"[All Fields] OR "predictiveness"[All Fields] OR "predictives"[All Fields] OR "predictivities"[All Fields] OR "predictivity"[All Fields] OR "predicts"[All Fields]) AND ("prevent"[All Fields] OR "preventability"[All Fields] OR "preventable"[All Fields] OR "preventative"[All Fields] OR "preventatively"[All Fields] OR "preventatives"[All Fields] OR "prevented"[All Fields] OR "preventing"[All Fields] OR "prevention and control"[MeSH Subheading] OR ("prevention"[All Fields] AND "control"[All Fields]) OR "prevention and control"[All Fields] OR "prevention"[All Fields] OR "prevention s"[All Fields] OR "preventions"[All Fields] OR "preventive"[All Fields] OR "preventively"[All Fields] OR "preventives"[All Fields] OR "prevents"[All Fields])) OR "precision prevention"[All Fields] OR "stratified prevention"[All Fields] OR "tailored prevention"[All Fields]) AND ("intervent*"[All Fields] OR "activit*"[All Fields] OR "approach*"[All Fields] OR "path*"[All Fields] OR "program*"[All Fields] OR "strateg*"[All Fields] OR "plan*"[All Fields]) AND ("genomic*"[All Fields] OR "epigenomic*"[All Fields] OR "metabolomic*"[All Fields] OR "transcriptomic*"[All Fields] OR "pharmacogenomic*"[All Fields] OR "radiomic*"[All Fields] OR "omic*"[All Fields])) AND (2017:2024[pdat])

References: 5.399 - queried on 29^th^ January 2025

**Search strategy for Scopus:**

ALL ( ( ( "personal* prevention" OR "individual* prevention" OR "predictive prevention" OR "precision prevention" OR "stratified prevention" OR "tailored prevention" ) AND ( intervent* OR activit* OR approach* OR path* OR program* OR strateg* OR plan* ) AND ( genomic* OR epigenomic* OR metabolomic* OR transcriptomic* OR pharmacogenomic* OR radiomic* OR omic* ) ) , ) AND PUBYEAR > 2016 AND PUBYEAR < 2025

References: 1.582 - queried on 29^th^ January 2025

**Search strategy for Web of Science:**

ALL=(((“personal* prevention” OR “individual* prevention” OR “predictive” OR “precision prevention” OR “stratified prevention” OR “tailored prevention”) AND (intervent* OR activit* OR approach* OR path* OR program* OR strateg* OR plan*) AND (genomic* OR epigenomic* OR metabolomic* OR transcriptomic* OR pharmacogenomic* OR radiomic* OR omic*)) (All Fields) and Article or Review Article or Publication With Expression Of Concern or Early Access (Document Types) and English (Languages) and Zoology or Agriculture or Pediatrics or Veterinary Sciences or Physics or Biophysics or Energy Fuels or Substance Abuse or Tropical Medicine or Biodiversity Conservation or History Philosophy Of Science or Entomology or Information Science Library Science or Electrochemistry or Microscopy or Archaeology or Geology or Oceanography or Allergy or Plant Sciences or Engineering or Mathematics or Forestry or Fisheries or Arts Humanities Other Topics or Construction Building Technology or Criminology Penology or Water Resources or History or Meteorology Atmospheric Sciences or Philosophy or Religion or Remote Sensing or Transportation (Exclude – Research Areas))

References: 12.862 - queried on 29^th^ February 2025

**Search strategy for grey literature:**

**Google and Google Scholar (first 100 results):**

(genomic OR genetic OR epigenomic OR epigenetic OR metabolomic OR transcriptomic OR pharmacogenomic OR radiomic OR omic) AND plan

(genomic OR genetic OR epigenomic OR epigenetic OR metabolomic OR transcriptomic OR pharmacogenomic OR radiomic OR omic) AND strategy

(genomic OR genetic OR epigenomic OR epigenetic OR metabolomic OR transcriptomic OR pharmacogenomic OR radiomic OR omic) AND program

(genomic OR genetic OR epigenomic OR epigenetic OR metabolomic OR transcriptomic OR pharmacogenomic OR radiomic OR omic) AND path

**Institutions and organisations websites (first 20 results):**

(genomic OR genetic OR epigenomic OR epigenetic OR metabolomic OR transcriptomic OR pharmacogenomic OR radiomic OR omic)

**List of organisations/institutions:**

| **Name of organisation/institution** | **Acronym** | **Country** | **Website link** |
| --- | --- | --- | --- |
| European Center for Disease prevention and Control | ECDC | Europe | https://european-union.europa.eu/index_it |
| G-BA – THE FEDERAL JOINT COMMITTEE | GBA | German | https://www.g-ba.de/ |
| HAUTE AUTORITÉ DE SANTè | HAS | France | https://has-sante.fr/ |
| European Medicine Agency | EMA | Europe | https://www.ema.europa.eu/en/homepage |
| International Consortium for Personalized Medicine | ICperMED | Europe | https://www.icpermed.eu/ |
| The Agency for Regional Health Care | AGENAS | Italy | https://www.agenas.gov.it/ |
| Centers for Disease Control and Prevention | CDC | USA | https://www.cdc.gov/ |
| World Health Organization | WHO | International | https://www.who.int/ |
| European Commission | EC | Europe | https://commission.europa.eu/index_it |
| BELGIAN HEALTH CARE KNOWLEDGE CENTRE | KCE | Belgium | https://kce.fgov.be/ |
| NATIONAL INSTITUTE FOR HEALTH AND CARE EXCELLENCE | NICE | United Kingdom | https://www.nice.org.uk/ |
| NATIONAL INSTITUTE FOR HEALTH AND CARE RESEARCH | NIHR | United Kingdom | https://www.nihr.ac.uk/ |
| NORWEGIAN INSTITUTE OF PUBLIC HEALTH | NIPH | Norway | https://www.fhi.no/ |
| SWISS FEDERAL OFFICE OF PUBLIC HEALTH | SFOPH | Swiss | https://www.bag.admin.ch/ |
| THE NETHERLANDS ORGANISATION FOR HEALTH RESEARCH AND DEVELOPMENT | ZonMw | Netherlands | https://www.zonmw.nl/ |
| Ministero della Salute | MdS | Italy | [www.salute.gov.it](http://www.salute.gov.it/) |
| Istituto Superiore di Sanità | ISS | Italy | [www.iss.it](http://www.iss.it/) |
| Società Italiana di Igiene | SItI | Italy | [www.sitinazionale.org](http://www.sitinazionale.org/) |
| Ministerio de Sanidad | MSCBS | Spain | <http://www.mscbs.gob.es/> |
| Ministère de la Santé et de la Prévention | - | France | [https://solidarites-sante.gouv.fr](https://solidarites-sante.gouv.fr/) |
| Ministry of Health, Welfare and Sport | - | The Netherlands | <https://www.government.nl/ministries/ministry-of-health-welfare-and-sport> |
| Global Burden of Disease | GBD |  | <https://www.healthdata.org/gbd> |
| European Public Health Association | EUPHA | Europe | <https://eupha.org/index.php> |
| Gesundheit Österreich GmbH | GÖG | Austria | [https://goeg.at](https://goeg.at/) |
| Austrian Agency for Health and Food Safety GmbH | AGES | Austria | <https://www.ages.at/en/> |
| European Health Information Portal | - | Europe | [https://www.healthinformationportal.eu](https://www.healthinformationportal.eu/) |
| Federal Ministry Republic of Austria | - | Austria | <https://www.sozialministerium.at/en.html> |
| Federal public service | FPS | Belgium | <https://www.health.belgium.be/en> |
| Belgian Association of Public Health | BAPH | Belgium | [https://www.baph.be](https://www.baph.be/) |
| Bulgarian Public Health Association | BPHA | Bulgaria | <https://baoz.bg/index.php/en/> |
| Croatian Public Health Association | HZJZ | Croatia | <https://www.hzjz.hr/en/> |
| German Public Health Association | DGPH | Germany | <https://www.dgph.info/en/> |
| Hungarian Association of Public Health Training and Research | NKE | Hungary | <https://nepegeszsegugyi-egyesulet.hu/en> |
| Slovenian Public and Environmental Health Professionals | SANITARC SI | Slovenia | [https://www.sanitarc.si](https://www.sanitarc.si/) |
| SOCIEDAD ESPAÑOLA DE SALUD PÚBLICA Y ADMINISTRACIÓN SANITARIA | SESPAS | Spain | [https://sespas.es](https://sespas.es/) |
| Society for Social Medicine & Population Health | SocSocMed | United Kingdom | [https://socsocmed.org.uk](https://socsocmed.org.uk/) |
| Faculty of Public Health | FPH | United Kingdom | [https://www.fph.org.uk](https://www.fph.org.uk/) |
| European Society for Medical Oncology | ESMO | Europe | [https://www.esmo.org](https://www.esmo.org/) |
| The Belgian Society of Medical Oncology | BSMO | Belgium | [https://www.bsmo.be](https://www.bsmo.be/) |
| Cyprus Oncology Society | OEK | Cyprus | [https://www.oncology-cy.eu](https://www.oncology-cy.eu/) |
| Hellenic Society of Medical Oncology | HeSMO | Greece | <https://www.hesmo.gr/en> |
| Associazione Italiana di Oncologia Medica | AIOM | Italy | [https://www.aiom.it](https://www.aiom.it/) |
| Latvian Society for Medical Oncology | LOKA | Latvia | <https://onkomed.lv/en/> |
| Sociedad Espanola de Oncologia Medicica | SEOM | Spain | [https://seom.org](https://seom.org/) |
| Association of Cancer Physicians | ACP | United Kingdom | [http://www.theacp.org.uk](http://www.theacp.org.uk/) |
| European Genome-Phenome Archive | EGA | Europe | [https://ega-archive.org](https://ega-archive.org/) |
| Centre fot Genomic Regulation | CRG | Europe | [https://www.crg.eu](https://www.crg.eu/) |
| Italian Istitute for Genomic Medicine | IIGM | Italy | <https://www.iigm.it/site/> |
| Genomics for Next Generation Healthcare | GenoMed4All | Europe | <https://genomed4all.eu/about/> |
| Genomics England | - | United Kingdom | [https://www.genomicsengland.co.uk](https://www.genomicsengland.co.uk/) |
| National Health Service | NHS | United Kingdom | [https://www.england.nhs.uk](https://www.england.nhs.uk/) |
| Sanger Institute | - | United Kingdom | [https://www.sanger.ac.uk](https://www.sanger.ac.uk/) |
| Personalized cancer therapy | - | USA | https://[personalizedcancertherapy.org/](http://personalizedcancertherapy.org/) |
| Genomic medicine Sweden | GMS | Sweden | https://genomicmedicine.se/ |
| Tervise Arngu Instituut | TAI | Estonia | https://www.tai.ee/en/personalisedmedicine |
| Centers for Personalized Medicine | ZPM | Germany | https://zpm-verbund.de/ |
| Nationales Netzwerk Genomische Medizin Lungenkrebs | nNGM | Germany | https://nngm.de/ |
| Navarra 1000 Genomes Project | NAGEN 1000 | Spain | https://navarrabiomed.es/ |
| Welsh Government services and information | - | Wales | https://www.gov.wales/ |

References: 1.389 - queried on 29^th^ January 2025
